# Wound healing in post-smolt Atlantic salmon (*Salmo salar* L.)

**DOI:** 10.1038/s41598-019-39080-x

**Published:** 2019-03-05

**Authors:** Lene Rydal Sveen, Gerrit Timmerhaus, Aleksei Krasnov, Harald Takle, Sigurd Handeland, Elisabeth Ytteborg

**Affiliations:** 1 University of Bergen, Postboks 7800, 5020 Bergen, Norway; 2 Nofima, Osloveien 1, 1430, Ås, Norway; 3 Cermaq Group AS, Dronning Eufemias gate 16,0102, Oslo, Norway; 4 Uni Research, Thormøhlens Gate 55, 5008 Bergen, Norway

## Abstract

Skin biopsies (5 mm) taken from behind the dorsal fin on Atlantic salmon post-smolts were followed over a 2 month period. The healing process was dominated by hemostasis, acute inflammation, and epidermal repair the first 14 days post wounding (dpw), as shown through imaging, histological evaluation, and transcriptomics. Most of the immune genes showed decreased expression after two weeks, approaching the levels of intact skin, as also reflected in sections where reduced inflammation in the wound bed was observed. Transcriptional events suggest recruitment of lymphocytes to the wound site during the acute phase, with activation of humoral responses from 14 dpw and onward. From the histology, a more adherent mucus was observed that correlated with altered transcription of glycosyltransferases. This may indicate different properties and functions of the mucus during the wound healing process. Wound contraction started between 14 and 36 dpw. The occurrence of these events was concurrent with granulation tissue formation, melanocyte migration and up-regulation of genes involved in extracellular matrix formation. The presented description of the wound healing processes in Atlantic salmon gives insight into comparative ulcerative biology in mammals and fish and provides both novel and updated knowledge that can be applied for improved best operational practices for fish welfare in aquaculture.

## Introduction

The skin of Atlantic salmon (*Salmo salar*), is a coherent and dense barrier that protects the interior of the fish against the outer environment. The skin covers the entire outer surface, including the head, fins and eyes. Lesions in the skin are a major economic problem for the farmer and a welfare issue for the fish^1^. There are two types of wounds, mechanically induced wounds and lesions caused by pathogens. Mechanical wound development is often associated with events that may cause skin damage such as handling, de-lousing, acute panic episodes, storms, and predators^1^. Pathogens that may cause wounds includes sea lice that feed on skin and mucus, and the winter ulcer bacteria *Moritella viscosa* and *Tenacibaculum* spp^1^. Most studies on skin problems in Atlantic salmon focus on the interactions between the skin and wound causing pathogens^2–5^. However, there is little knowledge about the actual wound healing process in Atlantic salmon skin without the presence of pathogens.

All the classical stages of wound healing: re-epithelialization, inflammation, cell proliferation with granulation tissue formation, and tissue remodeling, are conserved in fish^6^. The unique feature in both fish and amphibian wound healing is that they possess epithelial cells (keratocytes) that migrate from the intact surrounding tissue to cover the wound surface^7–10^. In Atlantic salmon, small superficial wounds may close within a few hours, but this re-epithelialization process is dependent on factors such as temperature, wound dimensions, stress, and nutrition^11–13^. Following re-epithelialization, a preliminary epidermis with mucus production is formed and this initial barrier protects the wound from the external environment^11,14,15^. Reconstitution of the epidermis is an important part of the healing process, but for deeper wounds, the dermis also needs to be regenerated. Since the dermis consists of several structures and cell types such as scales, bone cells, dense and loose connective tissue, fibroblasts, pigment cells, and capillaries^16^, complete regeneration depends on an orchestra of responses involving many cell types and tissues. This process is well studied in zebrafish, in which granulation tissue is already present at 2 days post wounding (dpw) and at 28 dpw the damaged skin is almost indistinguishable from intact tissue^6^. In contrast, the process of granulation tissue formation in salmonids is poorly understood. In salmonids, the formation of granulation tissue is reported to start between 10 and 42 dpw^12,17,18^, variations depending most likely on the methods used to assess the wounds, life stage, and temperature.

The aim of this study was to thoroughly investigate and describe the molecular processes involved in the different phases of dermal wound healing in post-smolt Atlantic salmon. The fish were wounded with a 5 mm punch biopsy tool, as previously described by several authors^11,17,19^, creating a full thickness wound on the dorsal flank of the fish. The advantage with full thickness wounds compared to other wound healing models such as scale loss and skin abrasion is that both dermal and epidermal repair may be studied, as well as wound contraction. By combining several methods, including photography, histology, immunohistochemistry, scanning electron microscopy and transcriptomics, important phases in the wound healing process in the skin of post-smolt Atlantic salmon was elucidated.

## Results

### Histology–early and late phase of repair

**Figure 1:**
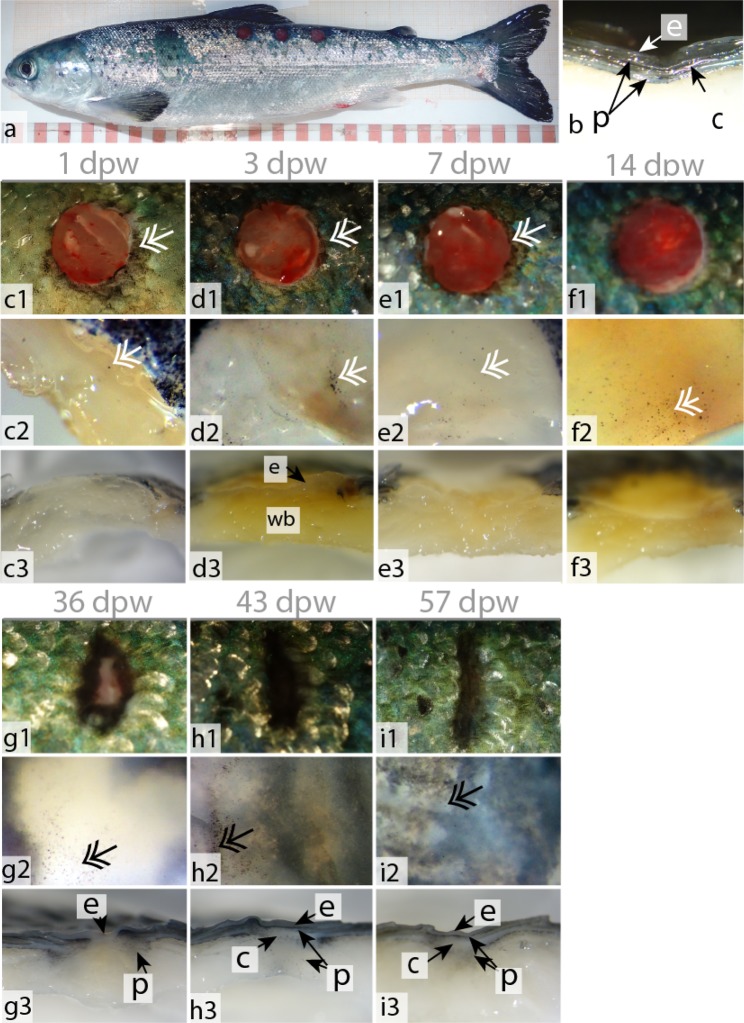
Wound contraction and pigmentation, during the early (c–f) and late (g–i)  healing phase. (**a**)  Fish with three punch biopsy wounds. (**b**) Stereoscope picture of intact skin with the epidermal layer (e), pigment cells (p) and collagenous tissue (c). (**c1–f1**) Photographs of the wounds during the early healing phase. Pigmented bodies at the wound margins (double arrow). (**c2–f2**) Stereoscope pictures (40×) of the wound surface. (**c3–f3**) Stereoscope pictures (16×) of horizontally cut wounds showing the wound bed (wb). From 3 dpw and onward it is also possible to see the epidermal layer covering the entire wounded surface. (**g1–i1**) Photographs of the contracting wounds during the late healing phase. (**g1–i1**) Stereoscope pictures (40×) of the wound surface. (**g3–i3**) Stereoscope pictures (16×) of horizontally cut wounds showing the epidermal layer, pigment cells and collagenous tissue. Photographs N = 12, stereoscope pictures N = 6. Columns represent dpw and rows different orientation and magnification of the wound.

Each fish was wounded with a 5 mm punch biopsy needle (Fig. 1a), and the wound healing process was followed intensively during the first week, with samplings at 1, 3 and 7 dpw and thereafter at 14, 36, 43 and 57 dpw. Images of unstained tissue samples showed a clear biphasic trend between early wound healing at 1–14 dpw and late wound healing 36–57 dpw. In the early healing phase, the wounds were red and open (Fig. 1c–f), while at 36–57 dpw the wounds were partly contracted and filled with a grey fibrous tissue (Fig. 1g–i), starting to resemble the structure of intact skin (Fig. 1b). Measurements of the wound width showed that the wounds expanded during the early healing phase. At 1 dpw the wounds had an average width of 6.5 (±0.04, SE) mm, and the wounds further expanded to an average width of 7.2 (±0.03) mm at 14 dpw. In the late healing phase the wounds were contracting. At 36 dpw the wounds had an average width of 3.9 (±0.03) mm and at the last sampling (57 dpw) the average width of the wounds was 2.9 mm (±0.06) mm. Each sampling point was further analyzed with a variety of histological techniques.

**Figure 2:**
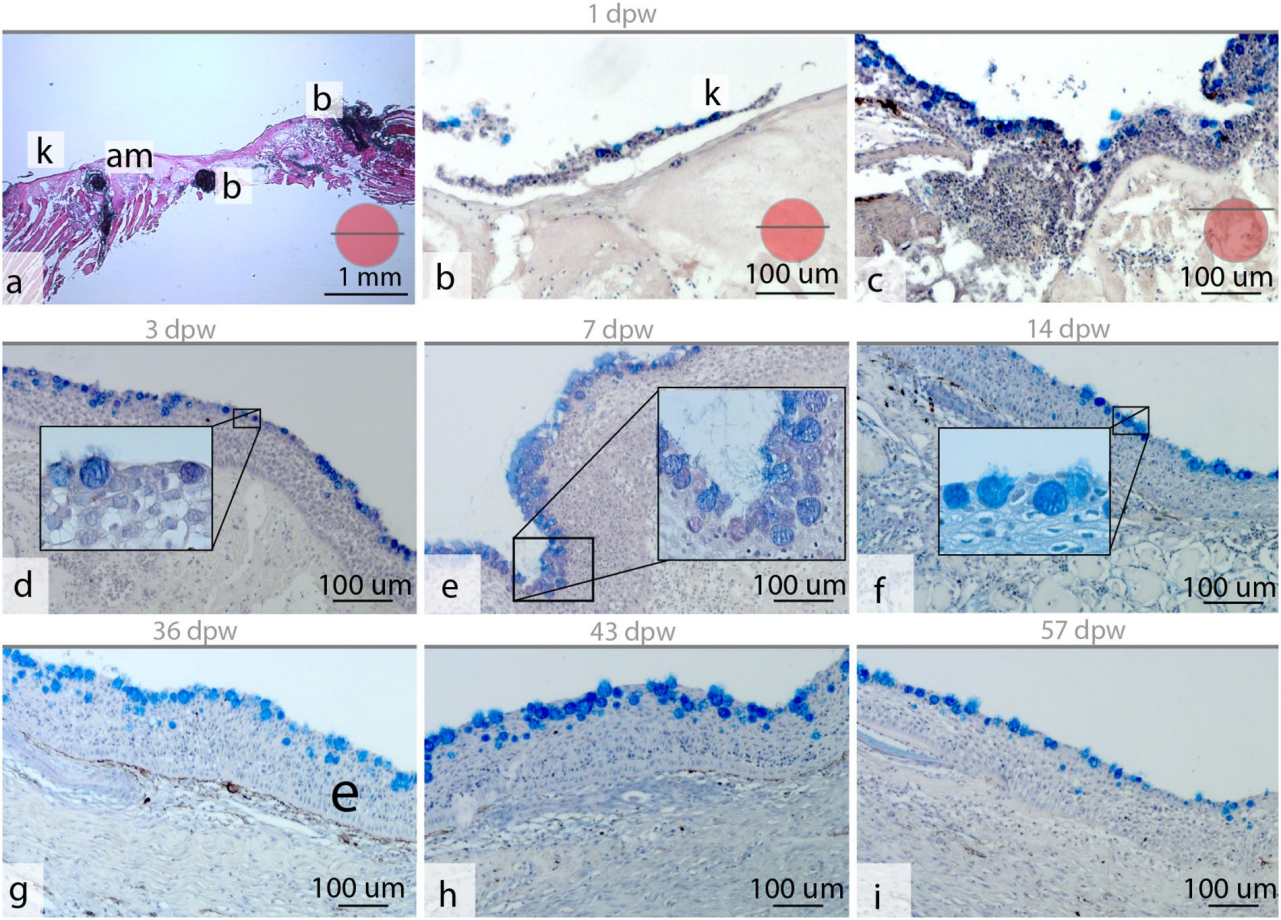
Epithelial mucus  response in the healing wounds. (**a**) Hematoxylin and eosin stained tissue section of the healing wound at 1 dpw, with general bleeding (b) and keratocytes (k) migrating on an amorphous substrate (am) (N = 4). (**b,c**) Alician blue and periodic acid-Schiff (AB/PAS) stained tissue sections showing mucous cells and keratocytes at the wound surface at 1 dpw (N = 4). (**d–i**) AB/PAS-stained tissue sections showing neutral (purple) and acidic (blue) mucous cells in the healing wounds at the respective time points (N = 3). The red circular insert figure indicates where the section was cut relative to the wound border.

**Figure 3:**
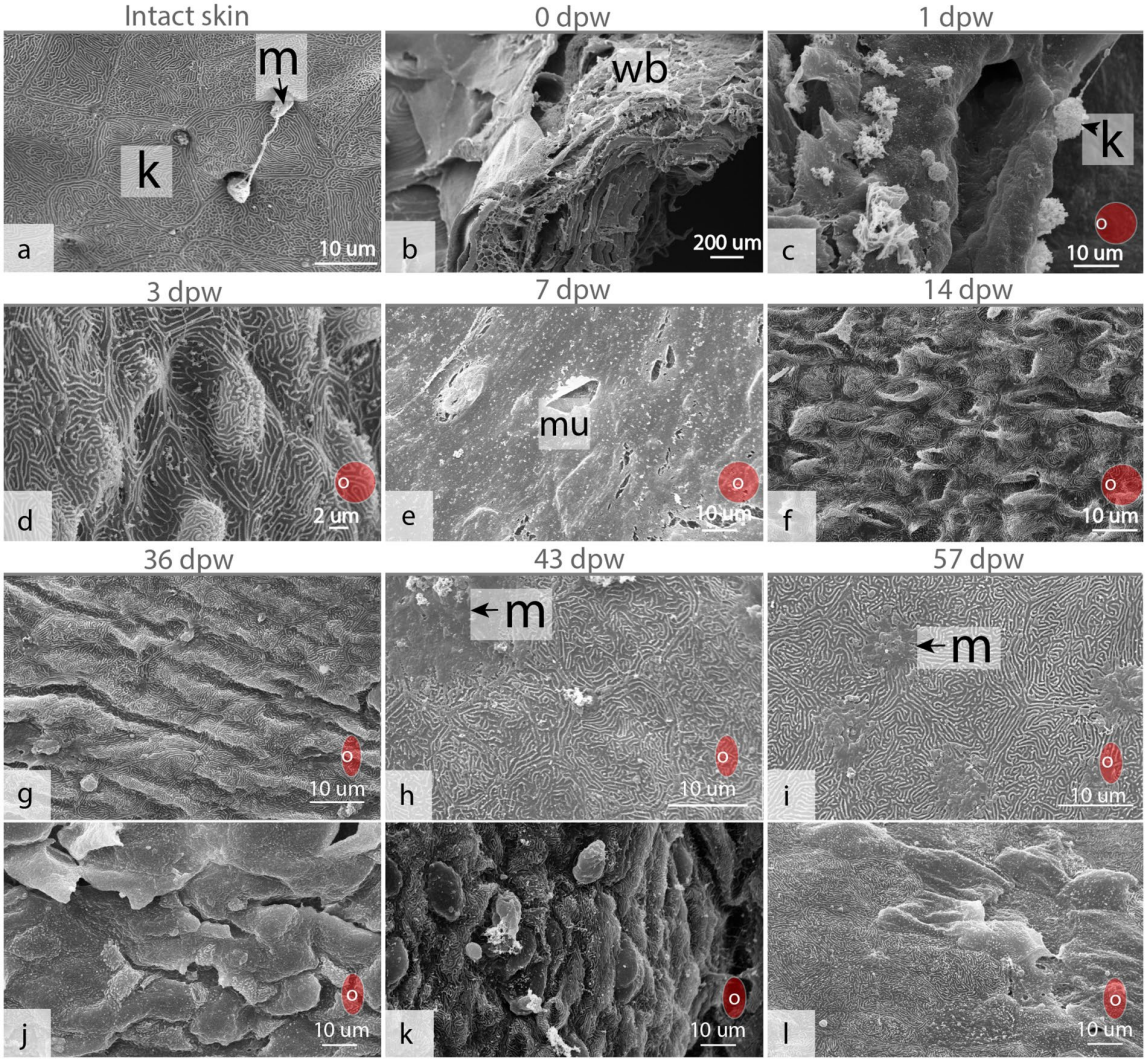
Scanning electron microscopy of the wound surface. (**a**) Intact skin, note the pentagonal shaped keratocytes (k) with their elevated microridge pattern, and protruding mucous cells (m). (**b**) The photo is taken directly after wounding showing damaged muscle fibers in the wound bed. (**c**) Keratocyte migrating on a smooth surface at 1 dpw. (**d**) Keratocytes at the surface with the typical microridge pattern at 3 dpw, note that the keratocytes are slightly elevated creating a rough surface. (**e**) A thick mucus (mu) gel covering the entire wounded surface at 7 dpw. (**f**) Keratocytes with protruding structures creating a rough appearance. (**g**) At 36 dpw the surface was folded at the wound edges  (**h,i**) Flat keratocytes and mucous cell at 43 and 57 dpw, the surface starts to resemble the surface structure of normal skin. (**j,k**) Structures in the wound center at 36, 43 and 57 dpw. N = 3 for all time points. The red circular insert figure indicates where the picture was taken relative to the wound borders.

At 1 dpw, the wound surface was fully or partly covered by an amorphous exudate (Fig. 2a), with migrating keratocytes at the wound margins (Figs 2a–c and 3a), and with general bleeding in the wound bed. Mucous cells were also found together with keratocytes at the migratory front (Fig. 2b). Near the wound border the several layers of keratocytes and plenty of mucous cells could be found (Fig. 2c).This was in contrast to the appearance of the wound directly after wounding, where damaged muscle fibers dominated in the wound bed (Fig. 3b). On the wound surface, pigmented bodies surrounded the wound margins causing hyperpigmentation (Fig. 1c1). These pigment bodies were found on the wound surface for the entire duration of the study (Fig. 1c2–f2), but were not observed on intact skin samples.

**Figure 4:**
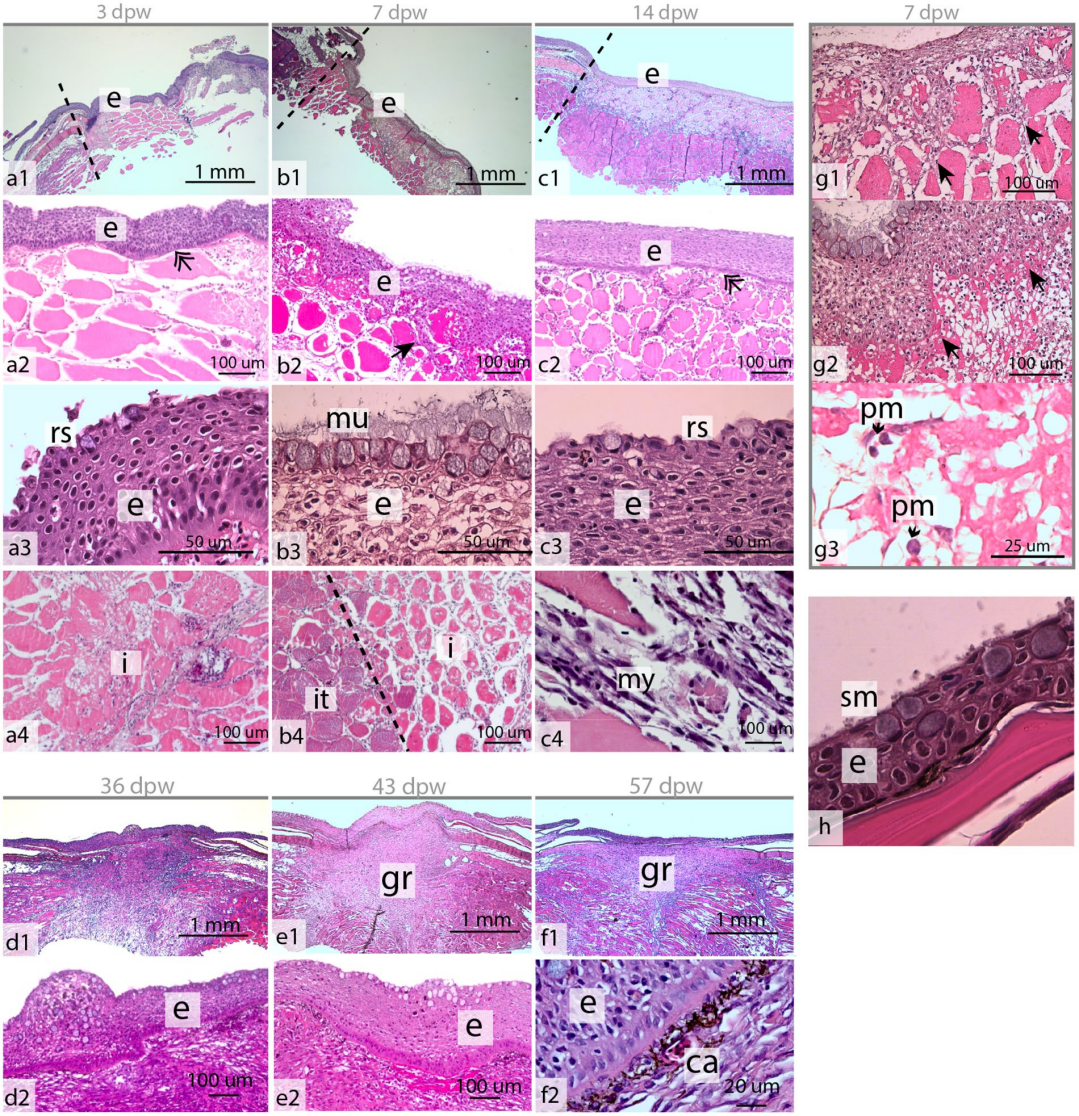
Hematoxylin and eosin stained tissue sections of the healing wounds during the early (a–c) and late (d–f) healing phase. (**a1–c1**) Overview of the healing wounds 3–14 dpw, with epidermal (e) layer and wound margin (black line). (**b1–b4**) The epidermal layer  at 3, 7 and 14 dpw. The epidermal membrane   was well defined at 3 and 14 dpw (double arrow), while keratocytes may be found together with damaged muscle fibers (solid black arrow) at 7 dpw. (**a3–c3**) Higher magnification of the epidermal layer. Note the rough surface (rs) of the superficial keratocytes at 3 and 14 dpw, and the strong mucus (mu) response at 7 dpw. (**a4–b4**) The wound bed with damaged tissue fibers. The border between intact (it) tissue and inflamed tissue is marked by a black, stippled line. (**c4**) Myofibroblast (my) like cells at 14 dpw. (**d1–f1**) Granulation tissue (gr) in the wound bed at 36–57 dpw. (**d2–f2**) The epidermal surface during the late healing phase, capillaries (ca) are present under the epidermal border at 57 dpw. (**g1,g2**) The border between the epidermal layer and the wound bed is not well defined at 7 dpw, each photo represents one individual. (**g3**) High magnification of b4 showing polymorphnuclear cells in the wound bed. **(h)** Epidermis in intact skin,  with smooth (sm) surface cells. 1–14 dpw (N = 4), 36–57 dpw (N = 3).

**Figure 5:**
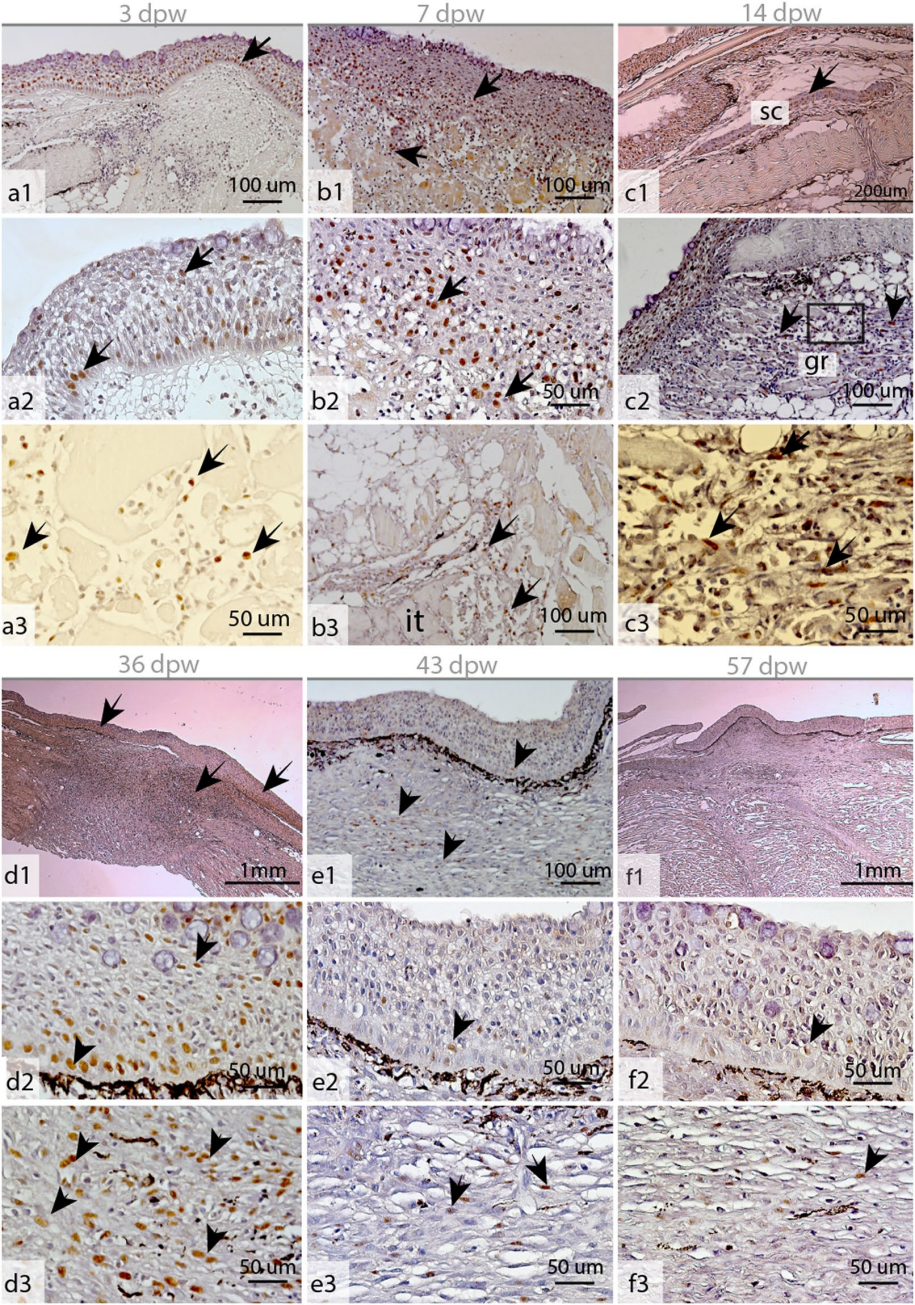
Proliferating cell nuclear antigen (PCNA) stained tissue sections of the healing wounds during the early (a–c) and late (d–f) healing phase. (**a1–c1**) Overview photos at 3, 7 and 14 dpw. (**a2,b2**) PCNA+ cells in the epidermal layer at 3 and 7 dpw. (**c2**) Granulation tissue with PCNA+ cells at 14 dpw. (**a3,b3**) PCNA+ cells in the wound bed at 3 and 7 dpw, these cells were located close to the intact (it) tissue. (**c3**) Higher magnification of the  rectangular area in (c2). (**d1–f1**) Overview photos of the healing wounds at 36, 43 and 57 dpw. (**d2–f2**) PCNA+ cells in the epidermal layer at 36, 43 and 57 dpw. (**d3–f3**) PCNA+ in the wound bed at 36, 43 and 57 dpw. Nuclei of PCNA+ cells stain brown (arrows). N = 3 for all time points.

At 3 dpw, a neo-epidermis covered the wound surface (Figs 1d3 and 4a1). The neo-epidermal layer had an organized appearance and was clearly separated by a membrane from the wound bed (Fig. 4a2). Scanning electron microscopy (SEM) of the wound surface showed that the keratocytes had developed their characteristic microridge pattern (Fig. 3d). However, the keratocytes on the surface were not flat as in intact skin (Fig. 3a), creating a smooth outer epithelial surface (Fig. 4h), but having elevated structures creating a rough surface (Figs 3d and 4a3). Further, mucous cells were only located in the superficial layer of the epidermis (Fig. 3d). Five percent of the mucous cells stained pink/purple with Alcian blue and periodic acid-Schiff (AB/PAS) staining (Table 1). Immunohistochemistry with proliferative cell nuclear antigen (PCNA) showed dividing (PCNA+) keratocytes in the entire epidermal layer (Fig. 5a1,a2). In the wound bed, a few PCNA+ cells were found at the wound margins (Fig. 5a3). However, the wound bed was dominated by damaged tissue fibers and infiltrating inflammatory cells (Fig. 4a4).

**Table 1:** Total number of mucous cells (blue + pink + purple) ± SEM, and percentage of pink and purple mucous cells compared to the total number of cells (N = 4 at 1–14 dpw, N = 3 at 36–57 dpw).

Days post wounding	3	7	14	36	43	57
Total number of mucous cells	61.4 ± 14.5	43.5 ± 12.5	26.5 ± 5.6	71.8 ± 12.7	56.8 ± 15.2	46.3 ± 4.4
Percentage pink/purple mucous cells	6.5 ± 2.7	24.0 ± 5.2	0	0.4 ± 0.4	1.2 ± 1.2	0.1 ± 0.5

The numbers were not significantly different according to Kruskal–Wallis rank tests.

At 7 dpw, the epidermal layer was no longer clearly separated from the wound bed and several keratocytes were found interspersed among the damaged muscle fibers (Fig. 4b2,g1,g2). These keratocytes were also PCNA+ (Fig. 5b1,b2). A strong cellular response with polymorph nucleated inflammatory cells dominated in the wound bed with vacuolization and degradation of muscle tissue (Fig. 4b4,g3). Several cells in the wound bed were also PCNA+. These cells were only found in the intersection between intact and damaged muscle tissue (Fig. 5b3). Further, a thick mucus layer covered the epithelial surface (Figs 2e, 3c and 4b3) and 25% of the mucous cells stained pink/purple with AB/PAS (Table 1).

**Figure 6:**
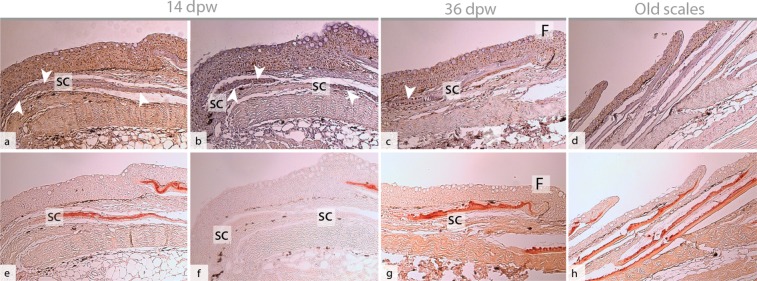
Proliferating cell nuclear antigen (PCNA) and Alizarin red staining of tissue sections, indicating scale formation at 14 dpw. (**a–d**) Tissue sections stained with PCNA, the nuclei of PCNA+ cells stain brown (arrowhead). (**e–h**) Tissue sections stained with Alizarin red, mineralized matrix stains red. The epidermal surface folds (F) around the scales (SC) as  they mature. Alizarin red N = 5 at 14 dpw and N = 3 at 36 dpw, PCNA N = 3 for both time points.

**Figure 7:**
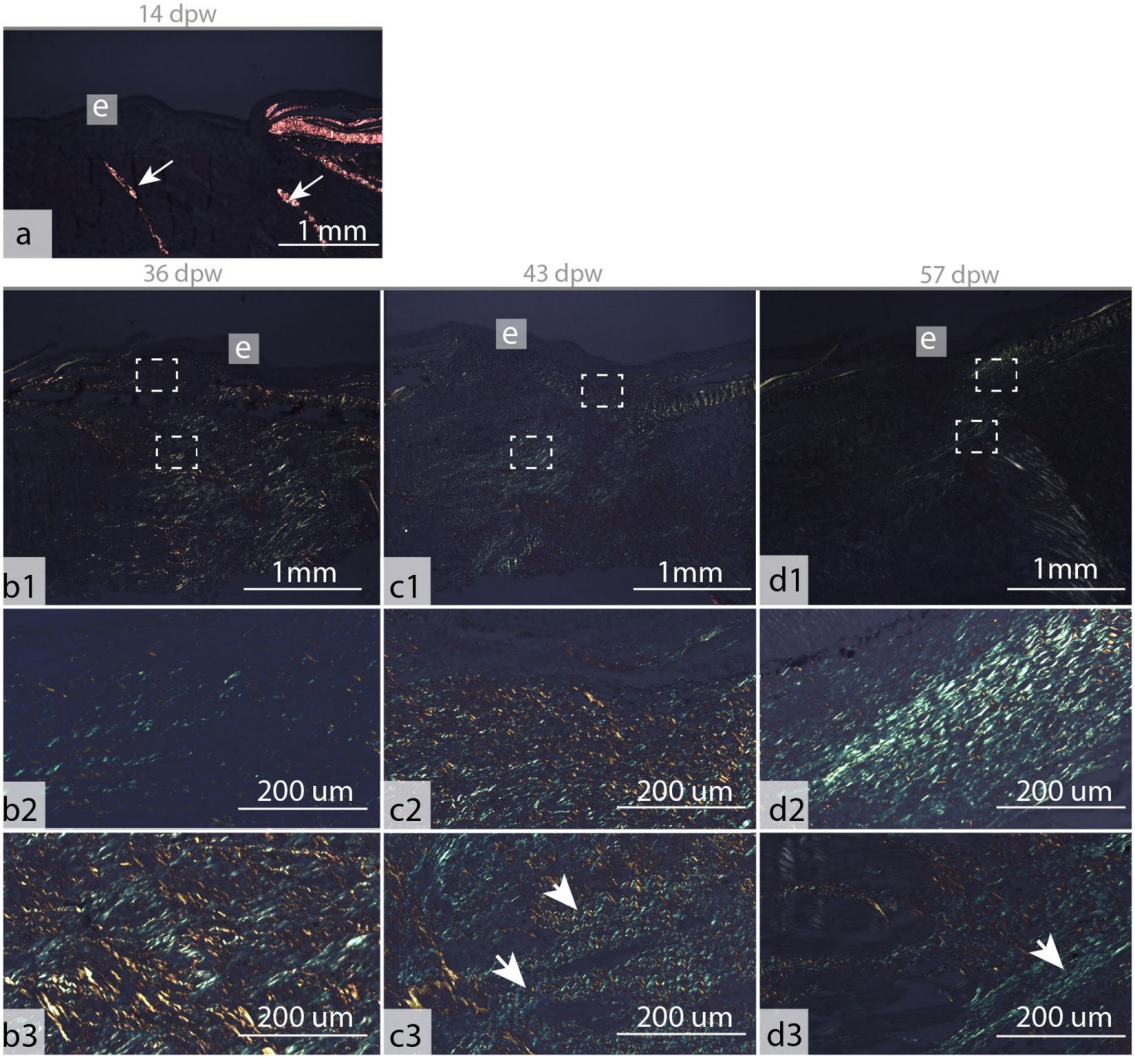
Picro sirius red stained tissue sections of the healing wounds at 14–57 dpw. (**a**) Collagenous structure in the healing wound at 14 dpw. (**b1–d1**) overview photos of collagenous structures in the healing wounds at 36–57 dpw. The epidermal (**e**) layer is indicated in the figure. (**b2**–**d2**) Collagenous structure beneath the epidermal surface, location as  indicated by the upper white rectangle in b1–d1. (**b3–d3**) Collagenous structure in the wound bed, location as indicated by the lower white rectangle in b1–d1. The photos were  taken under polarized light to differentiate the collagen fibres (red and green) from the background. Arrows indicate collagenous structures with horizontal orientations relative to the epidermal surface. N = 3 at all time points.

At 14 dpw, both inflammation and tissue repair were prominent in the wound bed. The epidermal layer was more organized compared to 7 dpw, with a clear separation to the wound bed (Fig. 4c1), however some keratocytes could still be found in the damaged muscle tissue (data not shown). On the wound margins, PCNA+ cells were organized in oval patterns on all samples (Figs 5c1 and 6). In contrast, “old scales” were not surrounded by PCNA+ cells (Fig. 6d). Staining for mineralized matrix with Alizarin red confirmed mineralized matrix in three out of five samples (Fig. 6e). New scales that did not contain mineralized matrix, were smaller in shape however they had a high number of PCNA+ cells (Fig. 6b,f). In the wound bed, granulation tissue was present at the wound margins (Fig. 5c2). In this tissue, several cells with elongated phenotypes, possibly myofibroblasts, were found (Figs 4c4 and 5c3). Newly formed collagen fibrils were also present in the wound bed. The collagen deposits were organized in rope-like structures protruding from the myocommata and into the wound bed (Fig. 7a). The wound surface was still rough (Figs 3f and 4c3). In the epidermal layer, blue mucous cells could be found at the surface (Fig. 2f) and less than 1% of the mucous cells stained pink/purple with AB/PAS (Table 1).

At 36 dpw, the wounds were partly contracted (Fig. 1g1), and filled with granulation tissue (Figs 4d1 and 5d1). The grey fibrotic tissue could also be visualized on macrophotographs (Fig. 1g3). Pigment cells, most likely melanocytes, were infiltrating the fibrotic tissue causing hyperpigmentation (Fig. 2e3).

Collagen deposits filled the wound bed, but the fibrils did not have a uniform direction (Fig. 7b1–b3). In addition, the wound bed was filled with plenty of PCNA+ cells (Fig. 5d1,d3). In the epidermal layer, PCNA+ cells were found at the wound margins, while very few PCNA+ cells were found in the center (Fig. 5d1). The epidermal layer was also thicker in the wound center compared to the surrounding regions (Fig. 4d2). The scales were more developed compared to the previous time points. The base was sinking into the dermis, while the epidermis folded around the anterior region of the scales (Fig. 6c,g).  SEM analysis showed that the epidermal surface was folded (Fig. 3g), maybe due to contractile forces in the wound bed. In the center of the wound there was an elevated wound ridge at this elevated “ridge” the keratocytes had round and elevated structures (Fig. 3j). Less than 1% of the mucous cells stained pink/purple with AB/PAS (Fig. 2g and Table 1).

At 43 dpw very few PCNA+ cells were found in the wound bed and in the epidermal layer (Fig. 5e1–e3). The collagen fibers had started to organize parallel to the epidermal layer, both in the wound bed and beneath the epidermal layer (Fig. 7c1–c3). Further, the melanocytes had migrated into two layers, one beneath the epidermal layer and one layer beneath the new collagenous tissue (Fig. 1h3). On the wound surface, the keratocytes close to the wound margins resembled that of normal skin with the cells obtaining flat pentagonal shapes with distinct microridge patterns (Fig. 3h). In the center of the wound, at the elevated wound ridge, the surface cells were taking on different elevated shapes, possibly being a combination of dead keratocytes in the process of detaching and newly recruited keratocytes about to differentiate into flat surface cells (Fig. 3k). Less than 1% of the mucous cells stained pink/purple with AB/PAS (Fig. 2h and Table 1).

At 57 dpw the wounds were fully contracted (Fig. 1i1). In the wound bed the collagen fibers beneath the epidermis were thicker with a more structured appearance compared to 43 dpw (Fig. 7d1–d3). Capillaries were also located directly beneath the epidermal layer (Fig. 4f2). In the wound bed and in the epidermis, very few cells were PCNA+ (Fig. 5f1–f3). On the wound surface, the wound ridge was still prominent, with the same features as described at 43 dpw (Fig. 3i,l). Less than 1% of the mucous cells stained pink/purple with AB/PAS (Fig. 2i and Table 1).

### Microarray

**Figure 8:**
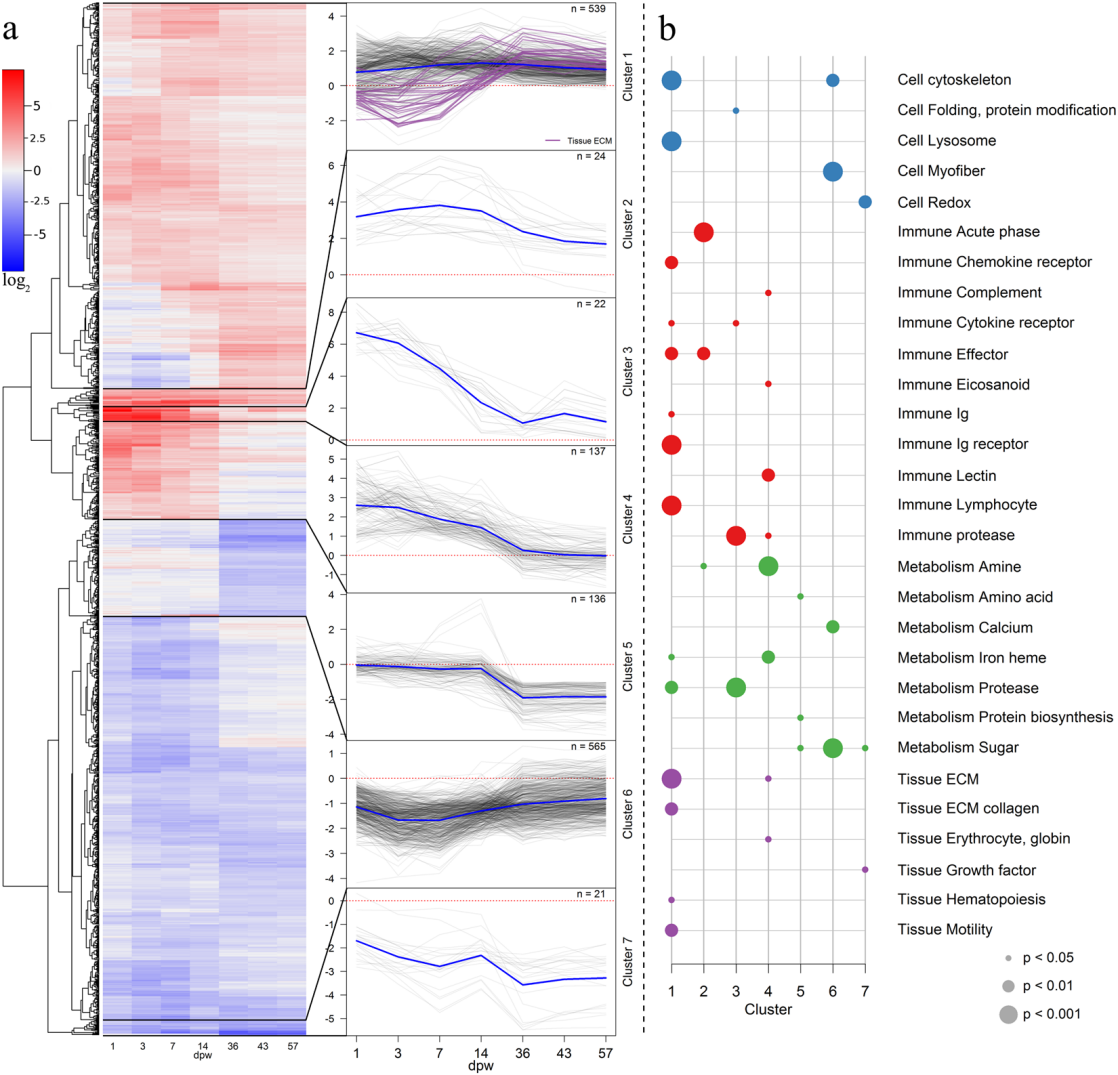
Transcriptomic responses to wounding. (**a**) The heatmap on the left shows transcription profiles of 1444 differentially expressed genes. The colors represent log_2_ ER relative to control, red for up-regulated and blue for down-regulated genes. The clusters to the right show the expression profiles for each gene within the respective cluster as a thin grey line. Blue lines represent the average within the cluster. Genes involved in extracellular matrix or collagen pathways in cluster 1 are marked with purple lines (**b**). Enrichment results for functional categories within the seven clusters. Blue for “cell”, red for “immune”, green for “metabolism” and purple for “tissue”. The sizes of the dots indicates the Fisher-test p-values (0.05, 0.01 and 0.001) of the enrichment analysis.

Wounding had a major impact on the transcriptomic response with >2000 differentially expressed genes (DEG) at three or more of the analyzed time points. Thus, clustering of DEG with known functions (N = 1444) was performed to reveal general trends in the transcriptomic profiles (Fig. 8). The majority of the DEGs in cluster 1, which contained 37% of the DEGs, were constantly up-regulated during the whole study and contained genes involved in cytoskeleton, lymphocyte, immune receptor and effector pathways. Within cluster 1 some genes  were also down-regulated during the first 14 dpw and up-regulated at later time points. These genes were involved in extracellular matrix and collagen pathways. Clusters 2 to 4 were strongly up-regulated at 1–14 dpw and contained DEGs of different immune pathways (Fig. 8b). The high expression levels typically weakened at later time points, yet some DEGs remained relatively high (Fig. 8a). These were proteases, immune acute phase genes, and effectors of the immune response. Clusters 5 and 7 contained down-regulated genes with a decreasing trend throughout the study and only a few pathways were significantly enriched. Cluster 6 was the largest with 39% of DEGs and contained constantly down-regulated genes with an increasing trend. Genes involved in cytoskeleton, myofiber, sugar, and calcium metabolism pathways were part of this cluster.

**Figure 9:**
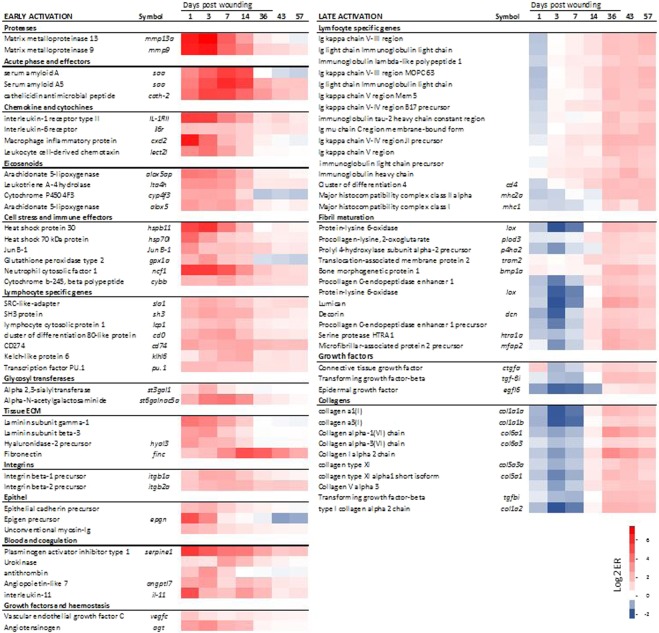
Transcriptomic response of selected genes. Selected genes representing the key early- and late-activated genes (left and right, respectively). Scale bar shows log_2_ ER relative to control, red for up-regulated and blue for down-regulated genes.

To understand the ongoing inflammatory and regenerative processes, we examined transcription of selected genetic markers activated in the early (1–14 dpw) or late (36–47 dpw) healing phases (Fig. 9). The matrix degrading proteases (*matrix metalloproteinase 9* and 13) were the most up-regulated genes in this study, with a log_2_ fold change of 7 (128-fold change) at 3 dpw (Fig. 9). The chemotactic factors for neutrophils and leukocytes (*leukocyte cell-derived chemotaxin, macrophage inflammatory protein*) also peaked in transcription between 1–3 dpw. Further, many genes with diverse immune functions and innate effectors, such as components of oxidative burst complex (*cytochrome b-245* and *neutrophil cytosolic factor*1), lipid signalling (*eicosanoids*) and multiple responders to cellular stress (*heat shock proteins*) and acute phase protein (*serum amyloids*) had a log_2_ fold change ≥2 (4-fold change) at 1–14 dpw. Genes known as activators of B and T cells (*SRC-like-adapter, SH3 protein*, *lymphocyte cytosolic protein 1* and *cd80-like protein*) and genes involved in B and T cell differentiation (*kelch-like protein 6, transcription factor PU.1* and *cd274*) stayed activated for the entire duration of the study (1–57 dpw).

Several genes involved in haemostasis and tissue repair also showed early activation (Fig. 9). These included genes involved in changes related to endothelial cell proliferation (*vascular endothelial growth factor*), endothelial migration (*angiopoietin*), proliferation of hematopoietic stem cells (*interleukin 11*), and vasoconstriction (*angiotensinogen*). At 1 dpw several genes involved in coagulation/anticoagulation (*antithrombin, urokinase, serpine1*) were also activated (Fig. 9), together with several genes involved in fibrin clot formation and extracellular matrix adhesion (f*ibronectin* and *integrins*). Further, several genes associated with epithelial migration (*epigen precursor, unconventional myosins*), cell to cell adherence (*epithelial cadherins*), and components of the basement membrane (*laminin* and *hyaluronidase-2*) were active at 1 dpw. Transcription of the glycosyltransferases *alpha 2,3-sialyltransferase* and *Alpha-N-acetylgalactosaminide-alpha-2,6-sialyltransferase6* increased during the early healing phase.

Late activation was shown for many genes involved in the synthesis of immunoglobulins together with antigen-presenting proteins such as the *major histocompatibility complex* −1 and −*2a* and *cluster of differentiation 4*, a key marker of T helper cells (Fig. 9). Other genes showing late activation were multiple collagens (*types I, IX, V, VI* and others) and proteins involved in maturation of fibrils. Late activation was also shown for several growth factors such as *transforming growth factor-beta*, *connective tissue growth factor* and *epidermal growth factor*.

## Discussion

**Figure 10:**
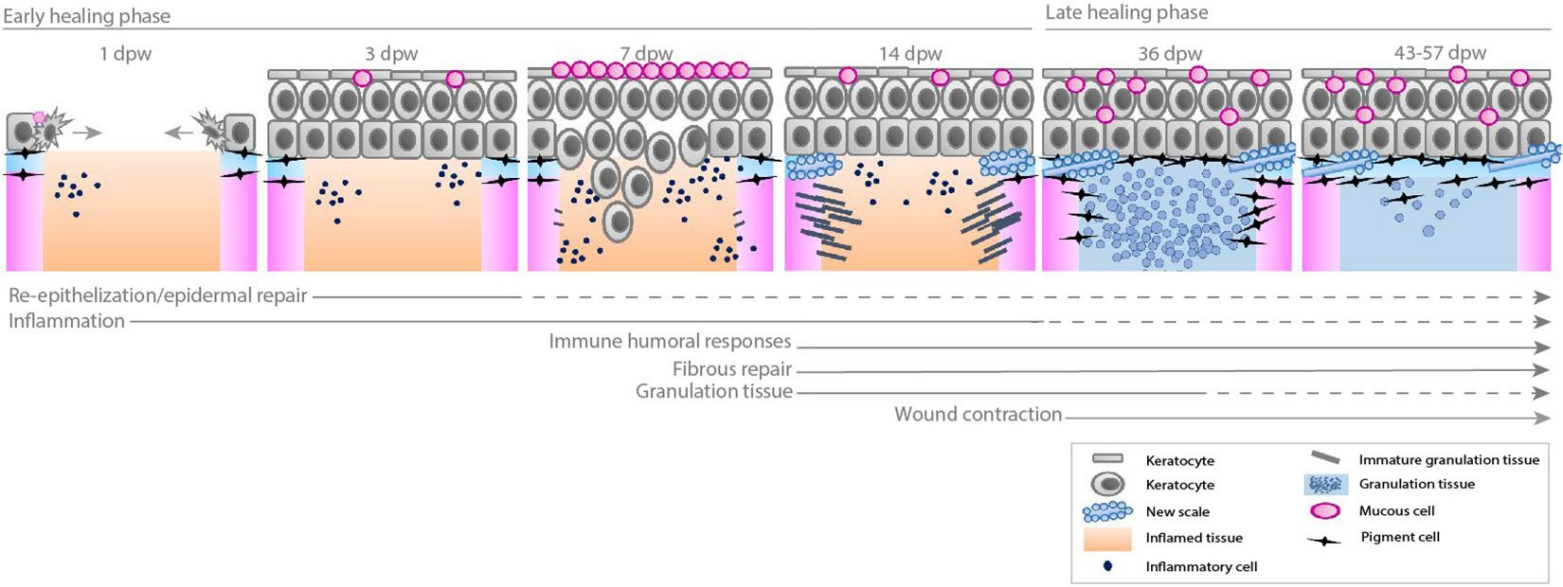
Summary of healing events of deep cutaneous wounds in Atlantic salmon post-smolts reared at ~10 °C. The wounds showed classic progression of healing: hemostasis, re-epithelialization, inflammation, tissue regeneration and remodeling. The early healing phase was dominated by hemostasis, epidermal repair, strong inflammation and tissue degradation. 14 dpw represented a transition point, as both inflammation (innate immunity/tissue degradation and onset of humoral responses) and fibrous tissue regeneration (scale formation, collagen deposition and granulation tissue formation) were active. The wounds started to contract between 14 and 36 dpw. At 36 dpw, granulation tissue filled the entire wound bed and pigment cells migrated into the fibrous tissue. From 43–57 dpw, granulation tissue was regressing, and the wounds were approaching the phenotype of normal skin.

We observed a clear separation between the early (1–14 dpw) and late (36–57 dpw) healing phases. The separation was manifested in the wound morphology (expanded vs. contracted), histology (inflammation vs. fibrous tissue) and gene expression data (inflammation vs. fibrous tissue repair). The early healing phase could be further separated into re-epithelization and hemostasis which was activated at 1 dpw and acute inflammation at 3 to 7 dpw, while 14 dpw represented a transition stage between acute inflammation and the onset of fibrous repair. In the late healing phase, proliferation and collagen deposition was prominent at 36 dpw, whereas tissue remodeling was established at 43–57 dpw. The timeline of the most important wound healing events in the skin of Atlantic salmon are summarized in Fig. 10.

At 1 dpw, re-epithelialization and hemostasis were activated. At the wound margins, both migrating keratocytes and mucous cells were found. Several genes involved in epidermal repair and hemostasis were up-regulated, such as *integrins* and *fibronectin*, which are used by platelets to mediate aggregation and fibrin clot formation^20,21^. In mammals, platelet activation also results in the release of a number of signaling molecules such as *vascular endothelial growth factor*^22^, and *connective tissue growth factor* that can induce sustained fibrosis^23^, these were also up-regulated immediately by wounding. In the gills of rainbow trout (*Oncorhynchus mykiss*), *eicosanoids* play a significant role in vasoconstriction^24^. As several genes involved in *eicosanoid* metabolism were up-regulated immediately by wounding, a similar role might be present in fish skin. Genes involved in blood coagulation such as *antithrombin*, *urokinase*, and *serpine1* were also up-regulated immediately by wounding. Since the absence of blood clot formation is considered a hallmark in the healing of fish skin^6^, these genes may have other functions in fish. Nevertheless, an amorphous exudate of unknown origin covered the wound surface at 1 dpw. A similar amorphous exudate has been reported in wounds of juvenile Atlantic salmon^12^ and in rainbow trout^18^. Thus, we assume the formation of fibrin-like structures would initially protect the wound surface and provide a smooth surface aiding epithelial migration. A novel finding in this study was the presence of mucous cells together with the migrating keratocytes at the wound borders at 1 dpw. This finding suggests that differentiation of mucous cells takes place as the keratocytes migrate on the wound surface. This contradicts findings in a previous study in which it was suggested that differentiation and proliferation of the keratocytes only occur when the migrating keratocytes cover the entire wound area^25^. The re-epithelization process was finished somewhere between 1 and 3 dpw, which is in concordance with previous research on 5 mm punch biopsy wounds in Atlantic salmon post-smolts^11^.

Wounding also led to immediate hyperpigmentation at the wound edges and spreading of pigmented bodies at the wound surface. These pigmented bodies were found on the wound surface at all investigated time points, but not on intact skin. Such a phenomenon is reported in several previous wound healing studies^26,27^. Given that melanin pigment has antioxidant activity and protects against environmental stressors^28–30^ and pathogens^31^, this pigmentation may be an initial mechanism protecting the wound surface.

Between 3 and 7 dpw, the histological analysis showed infiltration of inflammatory cells into the wound bed accompanied by tissue degradation. The transcriptomic results supported these findings and indicated the activation of an acute immune response and proteases from 1 dpw. This included transcription of immune effectors such as chemokines and interleukins that are involved in the recruitment of macrophages and other immune cells to the wound site. In general, the first two weeks post wounding showed a characteristic early innate immune response, similar to that observed in previous studies^32–35^.

At 7 dpw, histological analysis showed that there was no clear separation between the epidermal layer and the damaged muscle fibers. This might be a side effect of the inflammatory response and protease activity in the wound bed. The genes with the greatest overall change in expression ratio in this study were the *matrix metalloproteinases*. These proteases are secreted by both keratocytes and macrophages, and they are essential components of several parts of the wound healing process^36^. Further, they degrade extracellular matrixes such as laminins^37^, and fibrillar collagens, and they control inflammation by regulating the activity of cytokines and chemokines^38^. Thus, most of the observed tissue deterioration in the wound bed is likely linked to the high activity of these proteases and part of the natural wound healing process.

At 3 and 7 dpw, pink-purple stained mucous cells were present in the epidermis, whereas very few pink-purple mucous cells were found at the other time points. At 7 dpw, the percentage of acidic and neutral mucous cells was at the greatest abundance. At this time point, mucus was also sticking to the surface of SEM samples and to the AB/PAS samples. Indeed, for the SEM samples, the mucus gel completely covered the wounded surface, covering the epithelial cells. Being the only time point with these characteristics, increased adherence properties of the mucus gel is suggested. It has been shown that a large number of inflammatory mediators can directly enhance mucin transcription in mammals^39^, and alter the glycosylation pattern^40^, and the proportion of acidic and neutral mucins^41^. The observed alteration in the mucus response happened simultaneously with the high inflammatory response in the wound bed, and could possibly represent an additional epidermal protection mechanism.

At 14 dpw, both inflammation, granulation tissue, scale formation, and collagen fibrils were present in the wound. In this regard, 14 dpw represented the beginning of the transition between the early and late healing phase. The collagen structures were distinct, with three to four rope-like structures protruding from the intact muscle tissue. Given the distance between the rope-like structures, we suggest that fibroblasts may migrate from the myocommata, which are the major connective tissue compartment in teleost muscle^42^. At 14 dpw, myofibroblast-like cells were also present in the granulation tissue. In humans, these cells express α-smooth muscle actin, which plays a major role in wound contraction^38,43^.

The wounds started to contract between 14 and 36 dpw, thus the appearance of these cells may indicate the onset of wound contraction. Before 14 dpw, the wounds were expanding rather than contracting. Similar results are reported in rainbow trout^17^ suggesting that fish skin lacks a primary contraction mechanism. Such rapid contraction is a common feature of animals with loose skin including, mice, cats, dogs, and horses^44–46^. In humans and porcine, the dermis is firmly attached to the muscle and therefore primary wound contraction is limited^46,47^. Firm attachment of the muscle to the skin dermis is also the case in fish^42^. Therefore, wound contraction in salmonids is most likely driven by granulation tissue formation, similar to what is driving wound contraction in humans and porcine^47^.

At 14 dpw early scale differentiation with proliferating bone cells and deposition of mineralized matrix were present in the wounds. Osteoblasts proliferate as they differentiate from a mesenchymal stem cell to pre-osteoblast, thus the PCNA+ cells at 14 dpw indicate scale formation^48^. At 36 dpw there were few PCNA+ cells around the new scales, and the scales had features similar to late differentiated scales with epidermal folding in zebrafish^49^. In a parallel wound healing study, we recently showed that post-smolts reared at high fish densities had delayed scale mineralization^50^. In adult zebrafish both scales and pigmentation pattern completely recover after deep cutaneous wounding^6^, however a similar study in rainbow trout did not report scale regeneration during the healing of a 6 mm deep cutaneous wound^17^.

Further, a previous study following the healing of narrow incisional wounds in Atlantic salmon juveniles (reared at 10 °C) found that fibrous tissue repair starts at 14 dpw^12^. Here, we also report onset of fibrous repair at 14 dpw for 5 mm punch biopsy wounds. Thus, suggesting the possibility that onset of fibrotic repair may be independent to wound size and life-stage. In zebrafish, inflammation is important for both fibroblast recruitment and granulation tissue formation^6^, indicating fibroblast-stimulating signals from inflammatory cells in fish. This is also the case in mammals where macrophages promote granulation tissue formation and neo-vascularization^51^. Thus, inflammation is most likely needed to activate dermal repair in teleosts.

The late healing phase (36–57 dpw) was characterized by wound contraction and the formation of granulation tissue and scarring. At 36 dpw, an abundance of dividing cells was located in the wound bed, together with randomly distributed collagen fibrils. At 43–57 dpw, there were very few proliferating cells in the wound bed. The collagen fibrils were also more mature, being assembled into thicker structures aligned parallel to the epidermis. From 36–57 dpw, several genes involved in collagen synthesis, fibril maturation and growth factors such as *transforming growth factor-β* and *epidermal growth factor* were activated. Remodeling is the final phase of wound healing, and in this phase, the cell density and metabolic activity in the granulation tissue decreases. Changes also occur in the type, amount and organization of collagen, leading to an enhancement of the tensile strength of the tissues^36^. Given our findings, we suggest that  the wounds have entered the remodeling phase at 43 and 57 dpw.

The adaptive immune response was strongly activated from 36 dpw. In mammals, both the innate and adaptive immunity is required for wound healing^52,53^. Here we show that similar processes are likely occurring in fish. In mammals, the role of T-cells in wound healing is best described, with CD4+ cells suggested to enhance the wound healing process^54^, while other T-cell populations either have positive or negative effects on collagen deposits in the wounds^55–57^. B-cells also play a critical role in wound healing, as they may modify the immune cell populations and accelerate the healing process^58,59^. In this study, there was no indication of secondary infections in the wounds. Nevertheless, mechanical wounding will expose the tissue to an unsterile environment which is likely to also impact the immune response. In earlier trials with experimental virus infections of salmon smolts, we observed activation of B- and T-cell marker genes at 6 to 8 weeks post-infection^34,60^; thus, in the present study, the adaptive response was activated one or two weeks earlier compared to previous observations. Therefore, the results suggest that the activation of the adaptive immune response is connected to the wound healing and we suggest a role of activated lymphocytes in the fibrotic repair process in Atlantic salmon.

In the late healing phase, the epidermal layer was also under reconstruction. Proliferation of epidermal cells at the wound margins, together with few proliferative cells in the wound center, suggests a growth zone at the margins and a remodeling zone in the wound center. The proliferative activity in the epidermis decreased alongside with the proliferating activity in the wound bed. Analysis of the flat keratocytes at the wound surface also showed a different cell morphology of the cells located at the wound margins and those at the wound center. A similar appearance of flat keratocytes at the margins and elevated epithelial cells at the wound center has been reported in healing wounds of the teleost fish rohu carp (*Labeo rohita*)^61^. Thus, it appears that the epidermal layer at the edges of the wounds has a different function to the cells located at the wound center.

## Conclusion

The Atlantic salmon deep cutaneous wound model revealed an orchestrated wound healing process involving re-epithelialization, inflammation, innate and adaptive immune response, tissue repair and tissue remodeling, which is comparative to dermal wound healing in zebrafish. The detailed description of the wound healing processes in Atlantic salmon gives important insight for future targeted studies and practical information for operational fish health support and welfare considerations for fish farmers.

## Materials and Methods

### Experimental setup and sampling

This study was carried out at the Industrial Laboratory (ILAB, Bergen Norway) between November 4^th^, 2014 to January 30^th^, 2015. Smolts (mean size 80 g, N = 125) were reared in a single 500 L tank). During the experimental period, the mean fish density was 14 kg/m^3^. From the 4^th^ to the 6^th^ of November the fresh water in each tank was gradually replaced with seawater. The specific water flow was adjusted to 0.5 L/kg/min at the start of the experiment. The dissolved oxygen level in the outlet water was kept higher than 80% saturation by automatic oxygenation of the water in the header tanks (Oxyguard Commander). Both temperature (ranging from 9.4–10 °C) and oxygen saturation were measured daily (YSI 550, Xylem Inc., Yellow Springs, USA). Following transfer to seawater, the fish were exposed to a regime of 12 hours light and 12 hours dark. The fish were fed a commercial dry diet (EWOS, size 2–3 mm, Oslo, Norway).

On the 3^rd^ of December, skin biopsies were taken on 90 individuals with a 5 mm biopsy punch (Integra^TM^Miltex^TM^) as described by^11,17^. Prior to wounding, the fish were fully anesthetized with MS-222 (Sigma-Aldrich). Directly after wounding, the three layers of skin, epidermis dermis and hypodermis were removed, leaving a deep cutaneous wound with damaged muscle fibers. Samples (N = 12 fish per sampling point and group) were taken intensively during the first week (1, 3 and 7 dpw), and thereafter 14, 36, 43 and 57 dpw. Mortalities during the experiment period were low (<1%). Upon completion of the experiment, fish were euthanized with an overdose of anesthetic (MS-222). Samples for gene expression analyses were frozen immediately in liquid nitrogen and transferred to −80 °C for storage. The skin samples were fixed in a 4% paraformaldehyde solution (Electron microscopy science) overnight and then washed in 1 x PBS (Sigma Aldrich), before stepwise dehydration to 70% ethanol and finally transferred to −20 °C for storage. Skin samples for SEM were stored in PBS and not dehydrated.

### Imaging

Photographs of the fish and wounds were taken with a Sony Cyber-shot DSC-RX100 Zeiss camera (N = 12, for each time point). Unstained tissue samples were magnified under a Leica stereoscope (Leica wilde M3B), N = 6 for each time point, photos were taken through the optical lens with a Sony Cyber-shot DSC-RX100 camera.

Skin samples for histological evaluation were embedded in paraffin using a Leica TP1020 (N = 4 at 1–14 dpw and N = 3 at 36–57 dpw). Staining with Alcian blue and periodic acid-Schiff (AB/PAS) was done by staining the slides for 15 min in Alcian blue (Sigma-Aldrich), pH 2.5 followed by oxidizing the sections in 0.5% periodic acid solution (Sigma-Aldrich) for 5 min, followed by Schiff reagent (Merck) for 15 min and counterstaining in Mayer’s hematoxylin (Sigma-Aldrich) for 30 s Hematoxylin (Sigma-Aldrich) and eosin (Sigma-Aldrich) staining was done according to manufacturer’s instructions. Staining with alizarin red (Sigma-Aldrich) were done in a solution of 2 g Alizarin red in 100 mL dH_2_O pH 4.3 for 2 min. The Picro Sirius Red Stain Kit (Polysciences inc.) was used according to the manufacturer’s instructions. Staining of Proliferating Cell Nuclear Antigen (PCNA), was done with mouse anti-PCNA IgG2a (Millipore) and VECTASTAIN® Abc-HRP kit, anti-mouse IgG (Vector Laboratories) according to the manufacturer’s instructions (N = 3). Microscopy was carried out on a Zeiss Axio Observer Z1 (Carl Zeiss). Measurements were done in Aperio ImageScope (v12.3.2.8013). For evaluation of mucous cells (AB/PAS), a 10X objective was used. Mucous cells within the 10x magnification frame were counted. One frame approximately covered one-third of the wounded area at 1–14 dpw, and the half of the wound area at 36–57 dpw. The number of mucous cells was counted manually in two standardized areas on each sample, further differentiated between acidic (blue cells), neutral mucins (pink cells) and a mix of neutral and acidic mucous cells (purple cells).

Skin samples for SEM (N = 3) were dehydrated from PBS to absolute ethanol and dried using a Critical Point Dryer (CPD 030, Bal-tec AG, Schalksmühle, Germany) with liquid carbon dioxide as the transitional fluid. The samples were then mounted on stubs with carbon tape and coated with gold-palladium (Polaron Emitech SC7640 Sputter Coater, Quorum technologies, East Sussex, United Kingdom) and examined with SEM (EVO® 50 Series, Carl Zeiss AG, Oberkochen, Germany). Sample preparation and analysis were performed at the Imaging Center at the Norwegian University of Life Sciences.

### Transcriptomics

Tissue samples for quantitative real-time PCR (RT-qPCR) were stored at −80 °C prior to RNA extraction. Frozen skin sections (N = 6 per time point) were transferred directly to 1 mL chilled TRIzol (Thermo Fisher Scientific, Waltham, MA, USA) and homogenized in a Precellys®24 homogenizer. RNA was extracted from the homogenized tissues using a PureLink™ Pro 96 well purification kit (Thermo Fisher Scientific) with on-column-DNase (Qiagen, MD, USA) digestion according to the protocol for TRIzol-homogenised samples. The concentration of extracted total RNA was measured with a NanoDrop 1000 Spectrometer (Thermo Fisher Scientific) and RNA integrity was determined with an Agilent 2100 Bioanalyzer with RNA Nano kits (Agilent Technologies, CA, USA). Samples with an RNA integrity number (RIN) of 8 or higher were accepted.

Microarray analyses were performed using Nofima’s Atlantic salmon DNA oligonucleotide microarray SIQ-6 (custom design, GPL16555) containing approximately 15,000 probes of genes selected by annotations and expression profiles^62^. Microarrays were fabricated by Agilent Technologies. All reagents and equipment were purchased from the same source (Matriks, Oslo, Norway). All kits were used according to the manufacturer’s protocol. In brief, RNA amplification and labeling with Cy3 was performed with Low Input Quick Amp Labeling Kits (200 ng of total RNA per reaction) and Gene Expression Hybridization Kits were used for fragmentation of labeled RNA and preparation of the hybridization setup. Microarrays were hybridized for 17 h in a hybridization oven at 65 °C and rotation speed of 10 rounds per minute, washed for one minute with Gene Expression Wash Buffer I at room temperature, and one minute with Gene Expression Wash Buffer II at 37 °C. Washed slides were scanned with an Agilent SureScan Microarray scanner. Eight biological replicates from intact skin (control samples) and ten replicates per time-point were included in analyses, in total 38 arrays were used. Nofima’s bioinformatics package STARS^32^ was used for data processing and mining.

### Statistical analysis

For the microarray results, the differentially expressed genes were selected by the following criteria: log_2_-Expression Ratio >|0.8| (1.75-fold) and p < 0.05 relative to the un-wounded controls. A complete gene list of DEG, gene identifier and their respective STARS category can be found in Supplementary File 1. For the cluster analysis, t-tests relative to controls p < 0.01 and differential expression ±1 log_2_ fold change relative to controls were calculated for each gene and time point. Only genes significantly different from the controls at 3 or more time points were included in the analysis (1444 genes). The Euclidean distances were calculated, and the complete linkage clustering was drawn as a heat map. The dendrogram was pruned in order to identify 8 clearly-defined sub-clusters. One cluster contained only two genes and was thus excluded from the plot. Enrichment analyses of functional categories were calculated for each cluster with one-tailed Fisher tests to test for significant over-representation in the given cluster. Filtering, statistical analyses, and plotting of results were performed in R (version 3.3.1, www.r-project.org).

### Ethical statement

This study was approved by the local responsible laboratory animal science specialist under the surveillance of the Norwegian Animal Research Authority (NARA) and registered by the national ethics committee (the Norwegian Food Safety Authority) (ID7058). The methods were carried out in accordance with the relevant guidelines and regulations.

## Supplementary information

Supplementary Dataset 1

## Data Availability

Data were submitted to Gene Expression Omnibus (GSE122142).
